# Transgenerational Effects of Prenatal Ethanol Exposure in Prepubescent Mice

**DOI:** 10.3389/fcell.2022.812429

**Published:** 2022-03-21

**Authors:** Riley T. Bottom, Olga O. Kozanian, David J. Rohac, Michael A. Erickson, Kelly J. Huffman

**Affiliations:** ^1^ Interdepartmental Neuroscience Program, University of California, Riverside, Riverside, CA, United States; ^2^ Department of Psychology, University of California, Riverside, Riverside, CA, United States

**Keywords:** prenatal alcohol exposure, FASD, brain development, neocortex, behavior

## Abstract

**Background:** Fetal alcohol spectrum disorders (FASD) represent a leading cause of non-genetic neuropathologies. Recent preclinical evidence from suggests that prenatal ethanol exposure (PrEE), like other environmental exposures, may have a significant, transgenerational impact on the offspring of directly exposed animals, including altered neocortical development at birth and behavior in peri-pubescent mice. How these adverse behavioral outcomes are manifested within the brain at the time of behavioral disruption remains unknown.

**Methods:** A transgenerational mouse model of FASD was used to generate up to a third filial generation of offspring to study. Using a multi-modal battery of behavioral assays, we assessed motor coordination/function, sensorimotor processing, risk-taking behavior, and depressive-like behavior in postnatal day (P) 20 pre-pubescent mice. Additionally, sensory neocortical area connectivity using dye tracing, neocortical gene expression using *in situ* RNA hybridization, and spine density of spiny stellate cells in the somatosensory cortex using Golgi-Cox staining were examined in mice at P20.

**Results:** We found that PrEE induces behavioral abnormalities including abnormal sensorimotor processing, increased risk-taking behavior, and increased depressive-like behaviors that extend to the F3 generation in 20-day old mice. Assessment of both somatosensory and visual cortical connectivity, as well as cortical *RZRβ* expression in pre-pubescent mice yielded no significant differences among any experimental generations. In contrast, only directly-exposed F1 mice displayed altered cortical expression of *Id2* and decreased spine density among layer IV spiny stellate cells in somatosensory cortex at this pre-pubescent, post weaning age.

**Conclusion:** Our results suggest that robust, clinically-relevant behavioral abnormalities are passed transgenerationally to the offspring of mice directly exposed to prenatal ethanol. Additionally, in contrast to our previous findings in the newborn PrEE mouse, a lack of transgenerational findings within the brain at this later age illuminates the critical need for future studies to attempt to discover the link between neurological function and the described behavioral changes. Overall, our study suggests that multi-generational effects of PrEE may have a substantial impact on human behavior as well as health and well-being and that these effects likely extend beyond early childhood.

## Introduction

Fetal alcohol spectrum disorders, or FASD, is an umbrella term describing the group of clinical conditions that result from maternal consumption of alcohol, or ethanol, during pregnancy. In the United States alone, recent studies have reported 19.6% of pregnant women report first-trimester alcohol use (England et al., 2020) and that the prevalence of FASD may be as high as 5% in children (May et al., 2018). The adverse neurobehavioral effects of prenatal ethanol exposure (PrEE) present in patients with FASD are highly diverse and represent a leading cause of preventable intellectual disability worldwide (Lange et al., 2017). However, the neuropathology underlying these deficits is still not fully understood.

Animal models of FASD, as well as human patients, have helped scientists create hypotheses about developmental mechanisms that drive ethanol exposure-induced phenotypes in the central nervous system (CNS) and behavior. Within the CNS, several preclinical studies have shown multi-faceted aberrations in the development of the neocortex (Cuzon et al., 2008; El Shawa et al., 2013; Komada et al., 2017; Delatour et al., 2019, 2020; Mohammad et al., 2020), the brain region responsible for higher-order complex behaviors in mammals. Clinical studies in humans with FASD also report phenotypic variance in neocortex structure and function (Kodali et al., 2017; Hendrickson et al., 2018). Considering the cortical-associated behavioral disabilities in FASD, these data collectively support the notion that the neocortex may represent a focal point of ethanol’s teratogenic effects. The full range of neocortical developmental events that are possibly disrupted due to PrEE, how they manifest as neurological and behavioral impairments in later life, as well as the critical, driving mechanisms are still unclear.

Another important question concerning early ethanol exposure is this: Can PrEE induce heritable, transgenerational effects in offspring that are not directly exposed to ethanol via maternal drinking? Substantial, recent evidence has suggested that varied environmental exposures may produce transgenerational inheritance of disease phenotypes that are epigenetically-driven (Nilsson et al., 2018). Indeed, several recent reports have suggested that phenotypic variation induced by PrEE can extend several generations (Govorko et al., 2012; Gangisetty et al., 2020), likely via epigenetic mechanisms (Chastain and Sarkar, 2017). Because precise epigenetic regulation, or the control of gene expression not related to changes in DNA sequence, is also crucial for complex development of the neocortex (Adam and Harwell, 2020; Lewis et al., 2021) as well as how PrEE induces widespread epigenetic dysregulation in the brain (Subbana et al., 2014; Öztürk et al., 2017), the notion that underlying epigenetic mechanisms may be driving transgenerational FASD phenotypes represents an attractive hypothesis.

Recently, our laboratory described how distinct, PrEE-related neocortical phenotypes, including altered gene expression, methylation and neocortical connectivity, were observed across multiple generations of newborn mice after an initial prenatal exposure. Also, we showed that new behavioral phenotypes in peri-pubescent P30 mice, using the rotarod and forced swim test, extended to the F3 (third) generation after prenatal ethanol exposure in F1 (Abbott et al., 2018). Although some of these phenotypes mirror the human condition in FASD, the link between cortical abnormalities at birth and behavioral disruptions seen in later life warrants further investigation.

In this report, we extend our previous findings by assessing several multi-modal behavioral phenotypes that were not studied previously in P20 PrEE mice in the single or multi-generational FASD mouse models (El Shawa et al., 2013; Abbott et al., 2018). Using different behavioral tests, such as elevated plus maze and adhesive removal assays, we demonstrate robust behavioral disruptions at P20 that extend to the third generation after a single prenatal exposure to ethanol. These transgenerational PrEE-related phenotypes include altered sensorimotor processing, increased risk-taking behavior, as well as depressive-like behavior. Here, we also analyze cortical connectivity, gene expression and spine densities in somatosensory cortex in P20 mice, using our transgenerational mouse model of FASD (Abbott et al., 2018). Although neurobiological phenotypes were observed consistently at P0 and across generations in our prior work, it was unclear whether these phenotypes would persist past weaning, to age P20, which is analogous to middle childhood in humans. Our results show that gross CNS morphology and sensory neocortical connectivity abnormalities observed in newborn PrEE mice have recovered in all PrEE conditions by P20. However, abnormal gene expression and neocortical neuron spine density was present in directly-exposed F1 P20 PrEE mice. Specifically, F1 P20 PrEE mice had shifted *Id2* expression and reduced spine density in somatosensory spiny stellate cells at P20. Overall, our results suggest that substantial behavioral abnormalities are passed transgenerationally following PrEE and that the neurological changes driving these phenomena remain to be discovered. Outcomes from this study support the notion that the negative effects of PrEE are impactful beyond the directly exposed offspring and may require further consideration within humans.

## Materials and Methods

### Transgenerational FASD Mouse Model

**FIGURE 1:**
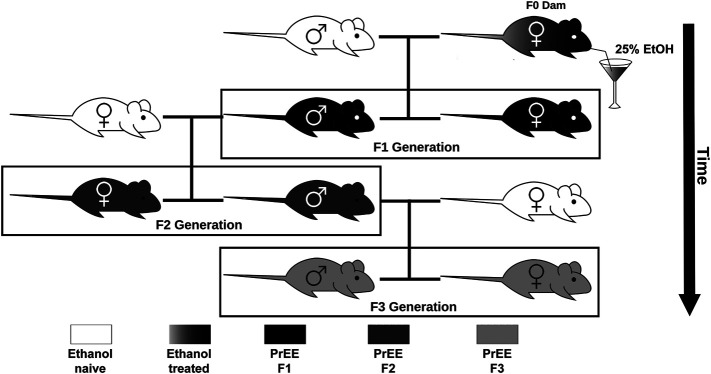
Transgenerational FASD model breeding paradigm. Initially, ethanol (EtOH) naïve P90 CD-1 mice were paired for breeding. Upon confirmation of copulation via vaginal plug, dams were given 25% EtOH in water throughout gestation (EtOH treated; gradient) to generate the prenatal EtOH exposed F1 experimental group (PrEE F1; black). Legend to 8–10 week old F1 males were then paired with EtOH naïve females to generate the PrEE F2 experimental group (dark gray). Lastly, legend to 8–10 week old F2 males were then paired with EtOH naïve females to generate the PrEE F3 experimental group (light gray).

All studies were conducted under a research protocol approved by the Institutional Animal Care and Use Committee at the University of California, Riverside. All control and experimental mice used in this study were of CD1 background, originally purchased from Charles River Laboratories International, Inc. (Wilmington, Massachusetts, United States) and were housed under normal illumination conditions (12 h light/dark cycle). For this study, we utilized a transgenerational FASD model previously established by our laboratory to generate control, F1, F2, and F3 subjects (Figure 1). Full details on transgenerational model are described elsewhere (Abbott et al., 2018). The male germline was used to produce subsequent generations following a single generation ethanol exposure. Briefly, 8–10 weeks old mice were paired for mating. Upon confirmation of vaginal plug, F0 dams were exposed to 25% ethanol in water or only water *ad libitum* throughout gestation. EtOH-exposed dams gave birth to directly-exposed F1 mice. Subsets of first filial generation (F1) males, once 8–10 weeks of age was reached, were bred with alcohol-naïve females to produce a subsequent F2 generation; this process was repeated, with F2 males bred with alcohol-naïve females, to produce a final F3 generation. Although potential heritability was passed through the male germline, both male and female offspring were used to study offspring phenotypes in all generations. The assurance of proper maternal nutrition in our transgenerational FASD mouse model including measures of weight gain, food and liquid intake, blood ethanol content, and blood osmolality has been previously documented (Abbott et al., 2018). Upon birth (P0), experimental (F1, F2, and F3) and control pups were cross-fostered with alcohol-naïve dams. Once mice reached P20, randomly chosen subsets of litters were subjected to behavioral testing, or were sacrificed and used for brain-based examination. For each experimental generation, control animals were included during testing. Pups included for analysis were limited to 1 per litter for brain-based analysis and 2 per litter for behavioral analysis to limit litter effects. All efforts were made to include near equal numbers of female and male per experimental endpoint to broadly assess the impact of transgenerational PrEE on both sexes. All experimental endpoint replicates per group (*n*) are reported in Tables 1, 2.

**TABLE 1:** Number of animals (n) in each experimental condition: behavior.

Measure	Sex	Control	F1	F2	F3
Accelerated Rotarod	Females	3	5	4	4
	Males	6	4	5	5
Adhesive Removal	Females	4	6	4	5
	Males	7	8	4	6
Elevated Plus Maze	Females	8	6	2	5
	Males	10	4	6	3
Forced Swim Test	Females	13	8	4	5
	Males	15	8	4	3

**TABLE 2:** Number of animals (n) in each experimental condition: anatomy.

Measure	Control	F1	F2	F3
Body weight	18	17	34	29
Brain weight	7	7	7	7
Cortical length	6	6	6	6
Dye tracing	8	8	6	6
ISH- RZR*β*	6	6	4	3
ISH- ID2	4	4	5	4
Spine density	14	17	12	15

### Brain Tissue Preparation and Measures

Following behavioral testing, PrEE F1, F2, F3 and control animals were given a lethal dose of sodium pentobarbital (100 mg/kg) and were transcardially perfused with 4% paraformaldehyde (PFA) in 0.1 M phosphate buffer (pH 7.4). Brains were then extracted from the skull, post-fixed in PFA for 24 h and hemisected. A separate subset of brains from each group was reserved for dendritic spine density analyses and were stored in 4% PFA for no longer than 2 months.

### P20 Pup and Brain Measures

At P20, prior to behavioral testing, all control, F1, F2, and F3 mice were weighed, using a standard Fisher Scientific scale to assess general development. After perfusion and dissection of P20 brain tissue, whole brains were weighed and dorsal views of whole brains were imaged using a digital high-resolution Zeiss Axio camera attached to a Zeiss Stereo Discovery V12 stereomicroscope using Axiovision software (Version 4.7). Lengths of neocortical hemispheres were measured in micrometers from the rostral pole just posterior to the olfactory bulb to the caudo-dorsal pole using the dorsal view images and an electronic micrometer in ImageJ (NIH). Detailed methods for cortical length measurements have been described in a previous report (Bottom et al., 2020).

### Anatomical Tracing Techniques and Analyses

Ipsilateral patterns of intraneocortical connections in control and PrEE F1, F2, and F3 P20 mice were examined by placing single DiI (1,1′-dioctadecyl-3,3,3′,3′-tetramethylinocarbocyanine perchlorate; Invitrogen, Waltham, MA, United States) and DiA (4-(4-(dihexadecyclamino)styryl-*n*-methylpyridinium iodide, Invitrogen, Waltham, MA, United States) crystals in somatosensory and visual cortices in a single hemisphere. Dye crystal placement reliability across experimental and control cases was promoted by using a dye placement grid in order to position crystals in a morphologically defined area. Following dye placement, hemispheres were re-immersed in 4% PFA and stored at room temperature for 8–12 weeks to allow for transport of the tracer. Control, F1, F2, and F3 hemispheres were then embedded in 5% low melting point agarose and sectioned in the coronal plane at 100 μm using a Vibratome. Sections were then counterstained with crystallized 4′,6-diamidine-2-phenylindole dihydrochloride (DAPI; Roche, Nutley, NJ, United States), mounted onto glass slides, coverslipped using Vectashield mounting medium for fluorescence (Vector Laboratories, Inc., Burlingame, CA, United States) and photographed with a digital high-resolution Zeiss Axio camera using Axiovision software (Version 4.7). Anatomical tracing sections were analyzed using a Zeiss Axio Imager Upright Microscope equipped with fluorescence at ×40 magnification. All fluorescent sections were digitally imaged three times for analysis of dye tracing experiments using three filters: blue for DAPI counterstain, red for DiI, and green for DiA labeling. Captured images were then combined in a high-resolution format for analysis. To analyze the effects of transgenerational PrEE on INCs at P20, sections from experimental and control brains were matched using anatomical landmarks. In depth tracing methodology has been previously described elsewhere (El Shawa et al., 2013; Abbott et al., 2018).

### Gene Expression Assays and Analysis

Standard non-radioactive free-floating *in situ* RNA hybridization methods (Dye et al., 2011a; Dye et al., 2011b; El Shawa et al., 2013) were used to visualize patterns of *RZRβ* and *Id2* expression in F1, F2, F3, and control neocortical tissue. Briefly, post-fixed hemispheres were embedded in gelatin-albumin and sectioned at 100 μm in the coronal plane using a Vibratome. Following hybridization to probes for *RZRβ* and *Id2* (gifts from John Rubenstein, UCSF), all sections were mounted in glycerol onto glass slides, cover slipped and photographed with a digital high-resolution Zeiss Axio camera using Axiovision software (Version 4.7). Both experimental and control sections were analyzed using a bright field on a Zeiss Stereo Discovery V12 stereomicroscope. Gene expression from ISH techniques was used as a qualitative measure for positional expression in F1, F2, F3, and control neocortex via side-by-side comparisons of expression levels in exact or near-exact anatomical levels of all brains by trained researchers blind to condition. Following qualitative analyses, ImageJ software (NIH) was used to statistically analyze *Id2* transcript positional levels between experimental groups. In order to quantify a visually identified medial shift in layer II/III *Id2* expression in caudal cortex (at the level of visual cortex) of experimental brains, the distance from the midline to the furthest lateral point of robust layer II/III *Id2* expression was measured in brains from all groups using an electronic micrometer in ImageJ.

### Spine Density Measurements

To assess the transgenerational effects of PrEE on dendritic spine development within the neocortex, a modified Golgi-Cox stain (GCS) protocol was used. Directly derived from two recent papers (Bayram-Weston et al., 2016; Zaquot and Kaindl, 2016), this modified GCS protocol was chosen for its ability to stain dendritic spines in extended post-fixed brain tissues (up to 2 months in 4% PFA). First, GCS stain was generated using two distinct solutions whose compositions are as follows: *Solution A.* 100 ml of 5% potassium dichromate solution (Sigma-Aldrich, no. P5271, United States) stirred into warm deionized water, with 100 ml 5% mercuric chloride (Sigma-Aldrich, no. M136, India) stirred into hot deionized water. *Solution B.* 200 ml of dH_2_O and 80 ml of 5% potassium chromate (Sigma-Aldrich, no. 216615, United States) stirred into cold deionized water. Solution A was then slowly poured into solution B with constantly stirring. Following correct mixing, a red-yellow precipitate was formed, completing the GCS.

Following GCS generation, hemisected brains from all four groups were placed into small, clean glass bottles that contained 15–20 ml of GCS. After 1 day, GCS was replaced with fresh solution. Brains remained submerged in GCS in the dark for 14 days. After 14 days, brains were briefly blotted and washed with PBS to remove excess GCS, then placed in 15–20 ml of 30% sucrose/0.1 M phosphate-buffered saline (PBS) in foil-covered container in 4°C until brains sank to the bottom (∼3 days). 30% sucrose/PBS solution was removed and replenished after 1 day of submersion. Following confirmation of sinking, hemispheres were embedded in 5% agarose and sectioned via Vibratome at 150 μm in the coronal plane. Sections collected in 30% sucrose/PBS were then mounted onto gelatin-coated “subbed” slides and were left to dry in racks in the dark for 2–3 days.

After drying, slides were developed by placing them into standard histological staining racks and submerging them through a series of solutions as follows: 1) Distilled water twice for 5 min each 2) 20% ammonia solution for 10 min 3) Distilled water twice for 5 min each 4) 70, 95, and 100% ethanol for 5 min each and 5) Xylene for 40 min. Immediately after removal from Xylenes, slides were cover slipped with Permount (Fisher Scientific, #SP15-500). Special care was taken to coverslip immediately due to rapid drying of sections following removal from Xylenes. Cover slipped slides were kept in horizontal slide box in the dark for 2 days prior to imaging.

Immediately before imaging, Golgi-Cox-stained sections were delineated for primary somatosensory cortex (S1) using anatomical landmarks. All cells within sections were imaged using brightfield microscopy at ×630 magnification using a Leica DFC 450C camera attached to a Leica Dmi8 microscope using Leica Application Suite software (version 4.6.0). Within S1, spiny stellate cells (confirmed via morphological characteristics) in layer IV were chosen to be imaged using random sampling. For S1 spiny stellate cell spine density measurements, a combination of primary and secondary branch dendrites was analyzed. For all groups, three mice were used for spine density measurement and an average of 4.833 cells were analyzed per mouse.

Following imaging, spine densities (spines/*μm*) for individual cells were calculated by manually counting all spines on a particular dendrite with a known dendritic length (as measured by an electronic micrometer in ImageJ). All manual spine counting was accomplished by a trained researcher blind to experimental condition.

### Behavioral Methods and Analyses

For all behavioral testing, mice were acclimated to the dimly-lit behavior room for at least 1 h prior to testing. All behavioral apparatuses were thoroughly disinfected and cleaned between subjects.

#### Accelerated Rotarod

The Accelerated rotarod is a behavioral assay that is used to measure motor coordination, balance and learning ability in mice (Pritchett and Mulder, 2003). The Rotarod apparatus is composed of a rotating rod separated into five lanes by four plastic dividers. On day of testing, mice were brought into the behavioral testing room in their home-cage and acclimated for 1 h prior to testing. Post-acclimation, both control and PrEE F1, F2, and F3 mice were tested on the rotating rod, which accelerates from 4–40 rpm over a 5 min trial period. Latency to fall from the bar was recorded for each animal during four consecutive 5 min trials, with each test trial being separated by a 30 min interval.

#### Adhesive Removal Test

The adhesive removal test is a method previously used in mice to assess sensorimotor deficits. Each animal’s somatosensory and motor function was evaluated by measuring the time needed to sense and remove adhesive tape strips from the snout (Fleming et al., 2013). The test mouse was scruffed by the experimenter, followed by the placement of an adhesive label onto the snout with the use of small forceps and released. Latency to remove the label with the forepaws was recorded. In the case where the mouse did not remove the sticker within 90 s, the trial was ended, and the experimenter removed the sticker manually. All mice received three trials, with each trial separated by a 10-min interval.

#### Forced Swim Test

The forced swim test is a common test used for evaluation of behavioral and neurobiological manipulations, particularly depressive-like states (Petit-Demouliere et al., 2005). The forced swim apparatus consisted of an acrylic glass cylinder, approximately 30 cm in height and 13 cm in diameter. Two-thirds of the cylinder was filled with room temperature water, with a video camera placed directly adjacent to the apparatus. All mice were assessed for a total of 6 min, however, the first 2 min of activity was considered an adaptive period, therefore only the last 4 min of the testing session were scored. All mice were scored by trained experimenters blind to condition for the total time spent immobile within the singular trial.

#### Elevated Plus Maze

The elevated plus maze test is a useful method typically employed to assess anxiety-related behaviors in rodents (Pellow et al., 1985). However, in young ages, CD-1 mice are known to defy these notions as they typically interact with the lower anxiety-associated open-arm regions at much higher proportions; this is thought to reflect risk-taking behavior (Macrí et al., 2002). The plywood elevated plus maze apparatus was elevated 50 cm above the floor and consisted of four arms, 54 cm wide and 30 cm long aligned perpendicularly. Two arms were enclosed by 15 cm high-walls and the other two arms were exposed. The maze was placed in the center of the behavioral testing room with a video camera located directly above it. Post-habituation, each animal was placed in the center of the elevated plus maze facing an open arm and left on the maze for a 5 min testing period. Time spent in the open arm, closed arm and center, as well as number of arm entries were scored from the video recordings.

### Statistical Analyses

Statistical analyses were performed using either GraphPad Prism 8 software (La Jolla, CA, United States) or R (Vienna, Austria). Group comparisons were performed using one-way or factorial ANOVA followed by Tukey’s multiple comparison post-hoc tests. For all statistical tests, a *p*-value of <0.05 was used to establish significance. Full *p* value summaries of all pairwise comparisons are shown in Table 3. All data are presented as mean ± SEM.

**TABLE 3:** *p* value summaries for all pairwise comparisons for each statistical test.

Statistic	F1-control	F2-control	F3-control	F2-F1	F3-F1	F3-F2
Adhesive Removal						
Mean time	0.0005	0.0064	0.0076	0.9869	0.8672	0.9851
Accelerated Rotarod						
Trial 1	0.0011	0.0469	0.0649	0.4440	0.3622	0.9988
Trial 2	0.0000	0.0023	0.0275	0.4383	0.0778	0.7493
Trial 3	0.4592	0.7213	0.8597	0.9720	0.8967	0.9934
Trial 4	0.7276	0.8051	0.8205	0.9990	0.9982	1.0000
Elevated Plus Maze						
Time in open arms	0.0017	0.6754	0.6933	0.1171	0.1109	1.0000
Forced Swim Test						
Time immobile	0.0000	0.0002	0.0013	0.9530	0.7036	0.9629
Pup Measures						
P20 body weight	0.0009	0.9997	0.1685	0.0003	0.0000	0.0742
P20 brain weight	0.0084	0.9978	0.9751	0.0128	0.0221	0.9953
P20 cortical length	0.0045	0.8733	0.9999	0.0242	0.0053	0.9001
Brain Measures						
P20 Id2—distance from medial wall	0.0217	0.4861	0.3322	0.1978	0.3953	0.9776
P20 spiny stellate cell—spine density	0.0016	1.0000	0.6662	0.0032	0.0000	0.6605

Note: Cells containing 0.0000 indicate *p* < 0.0001, and cells containing 1.0000 indicate *p* > 0.9999.

## Results

### Ethanol-Induced Transgenerational Effects on Complex Animal Behaviors

**FIGURE 2:**
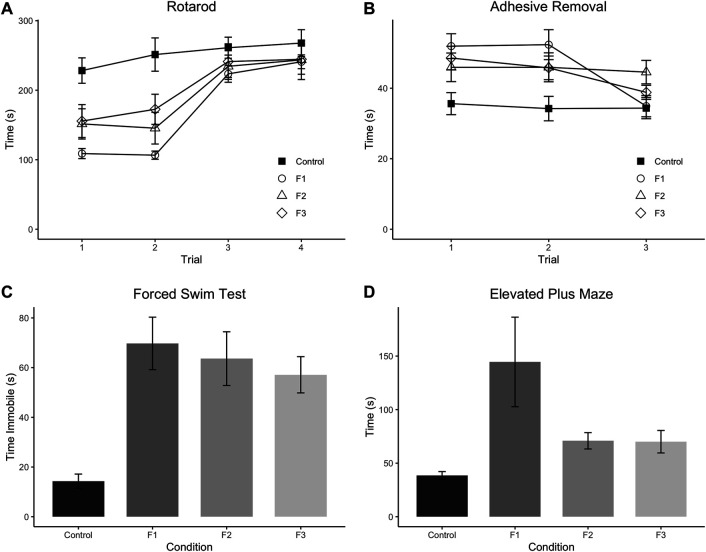
Multi-modality behavioral analysis. Results from behavioral testing in P20 control, F1, F2, and F3 mice in Accelerated Rotarod **(A)**, Adhesive Removal test **(B)**, Elevated Plus Maze **(C)**, and Forced Swim Test **(D)**. F1 mice display significantly decreased scores on trials 1 and 2 of the Rotarod compared to controls, and F2 mice only show significantly decreased scores on Trial 2 compared to controls. All three experimental generations display significantly increased scores on average across the three trials of the Adhesive Removal test compared to controls. F1 mice display significantly increased time spent on the open arms of the Elevated Plus Maze compared to controls, and all 3 PrEE generations display significantly increased time spent immobile compared to controls in the Forced Swim Test.

Initially, F1, F2, and F3 mice were assessed for ethanol-induced behavioral effects in several distinct modalities at P20, a prepubescent age. First, motor function/coordination and learning were examined via the Accelerated Rotarod, where all mice were given four trials spaced 30 min apart within a single day of testing (Figure 2A). A 3-way (sex x treatment x trial) repeated-measures ANOVA revealed a significant effect of treatment [F_3,28_ = 5.1340, *p* = 0.0059], and a significant trial × treatment interaction [F_9,84_ = 2.3533, *p* = 0.0202] in the Rotarod test. Post hoc tests revealed a significant decrease within trial 1 of F1 and F2 mice compared to controls (control: 228.28 ± 18.29 s, F1: 108.87 ± 7.23 s, *p* = 0.0011; F2: 151.55 ± 21.76 s, *p* = 0.0469) as well as a significant decrease for F1, F2, and F3 mice compared to controls in trial 2 (control: 251.31 ± 23.96 s, F1: 106.57 ± 5.92 s, *p* < 0.0001; F2: 145.40 ± 22.82 s, *p* = 0.0023; F3: 172.57 ± 21.68 s, *p* = 0.0275). However, F1 and F2 mice did not differ significantly from controls in trials 3 or 4, and F3 mice showed no significant differences in trials 1, 3, or 4, suggesting that motor coordination and motor learning dysfunction may be partially recovered by the third filial generation, reducing short-term motor learning impairments.

Next, sensorimotor processing and fine motor skills were examined via the Adhesive Removal task (Figure 2B), where mice were given three trials, spaced 10 min apart within a single testing day. A 3-way (sex x treatment x trial) repeated-measures ANOVA showed a significant effect of treatment [F_3,36_ = 5.0293, *p* = 0.0052] for this task. Post hoc analyses displayed a significant increase in time spent removing the adhesive in F1, F2 and F3 mice compared to controls across the three trials (control: 34.43 ± 1.73 s, F1: 46.39 ± 2.03 s, *p =*0.0005; F2: 45.57 ± 2.64 s, *p =* 0.0064; F3: 44.38 ± 1.38 s, *p =* 0.0076) suggesting that PrEE-induced perturbed sensorimotor processing and fine motor skill are passed transgenerationally.

Depressive-like behavior was evaluated via the Forced Swim Test (Figure 2C), and a significant effect of treatment was found on the time spent immobile within this test via a 2-way (sex x treatment) ANOVA [F_3,52_ = 8.4270, *p* = 0.0001]. Post hoc tests revealed a significant increase in time spent immobile in all three experimental generations compared to controls (control: 14.29 ± 2.88 s, F1:69.75 ± 10.56 s, *p <* 0.0001*;* F2:63.62 ± 10.80 s, *p =* 0.0002; F3:57.12 ± 7.30, *p =* 0.0013), suggesting increased depressive-like behavior due to PrEE that extends to the 3rd filial generation.

Lastly, anxiety-like and risk-taking behavior was also assessed in all three experimental groups at P20 via the elevated plus maze (Figure 2D). A 2-way (sex x treatment) ANOVA revealed a significant effect of treatment on time spent in the open arms of the maze [F_3,36_ = 3.7805, *p* = 0.0186], and post hoc tests indicated that only the F1 group showed a significant increase in time spent in the open arms compared to controls (control: 38.56 ± 3.60 s, F1: 144.54 ± 41.80 s, *p =*0.0017), suggesting that the normally elevated levels of risk-taking behavior in CD-1 mice on this task (Macrí et al., 2002) are even greater in mice directly-exposed PrEE mice. In summary, multi-modality behavioral testing revealed critical impairments in several complex behaviors in prepubescent mice, including several that are passed transgenerationally following single generation PrEE.

### Effects of Transgenerational Ethanol Exposure on Body and Brain Development

**FIGURE 3:**
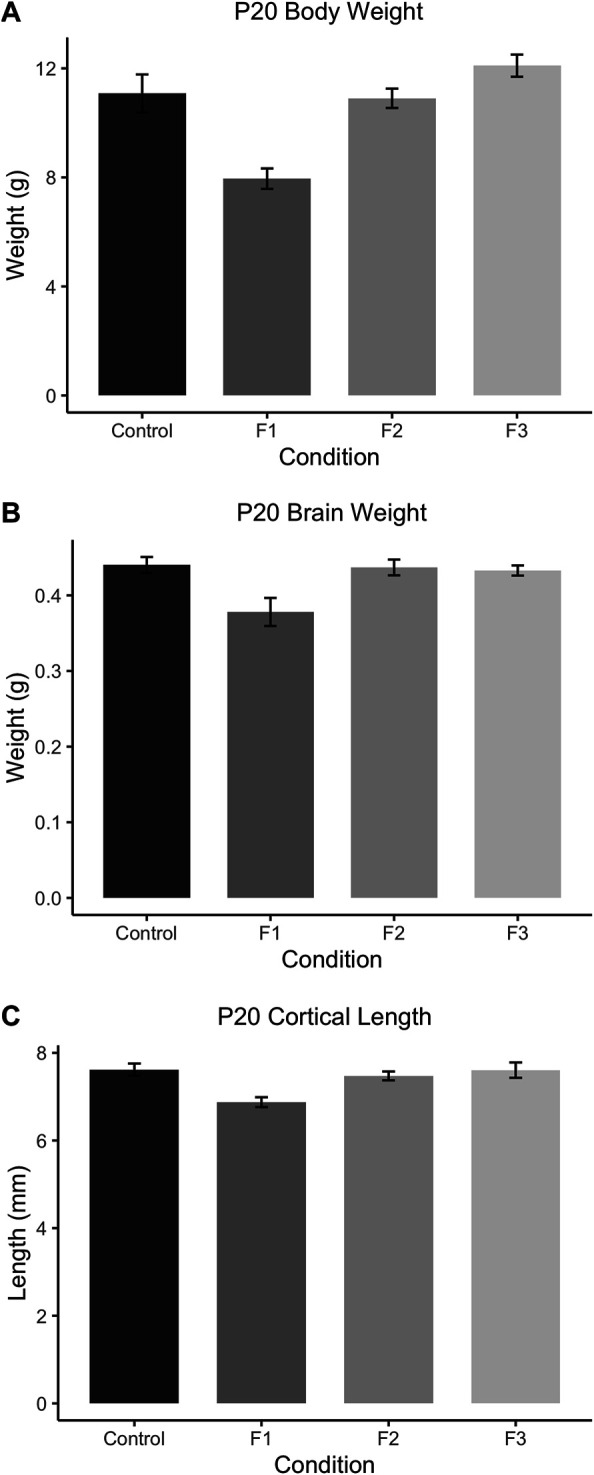
Gross body and brain measurements in P20 mice. **(A)** F1 mice weigh significantly less than controls at P20. P20 F1 mice also have significantly decreased brain weights **(B)** and cortical lengths **(C)** compared to controls.

To assess how PrEE affects body and brain development transgenerationally, body weights, brain weights, and cortical lengths were measured in P20 mice across F1, F2 and F3 generations and control mice (Figure 3). One-way ANOVA analysis showed a significant effect of treatment on P20 body weight [F_3,39_ = 12.73, *p* < 0.0001], and post hoc tests revealed a significant reduction in F1 body weight compared to controls (Figure 3A; control: 11.08 ± 0.6999 g, F1: 7.953 ± 0.3754 g, *p* = 0.0004d). A significant effect of treatment was also present in both brain weight [F_3,24_ = 5.565, *p* = 0.0045] and cortical length [F_3,20_ = 6.808, *p* = 0.0024] measurements at P20 (Figures 3B,C). Similar to P20 body weights, post hoc tests revealed only a reduction in the F1 generation for both brain weight (Figure 3B; control: 0.4004 ± 0.0107 g, F1: 0.3780 ± 0.0186 g, *p* = 0.0084) and cortical length (Figure 3C; control: 7.619 ± 0.1385 g, F1: 6.875 ± 0.1125 g, *p* = 0.0045) compared to control animals. Overall, these results suggest that in prepubescent mice, only the directly-exposed F1 generation display any significant effects of PrEE on overt body and brain development, where the cortex is particularly susceptible to PrEE’s effects.

### Assessment of Cortical Connectivity of Sensory Areas

**FIGURE 4:**
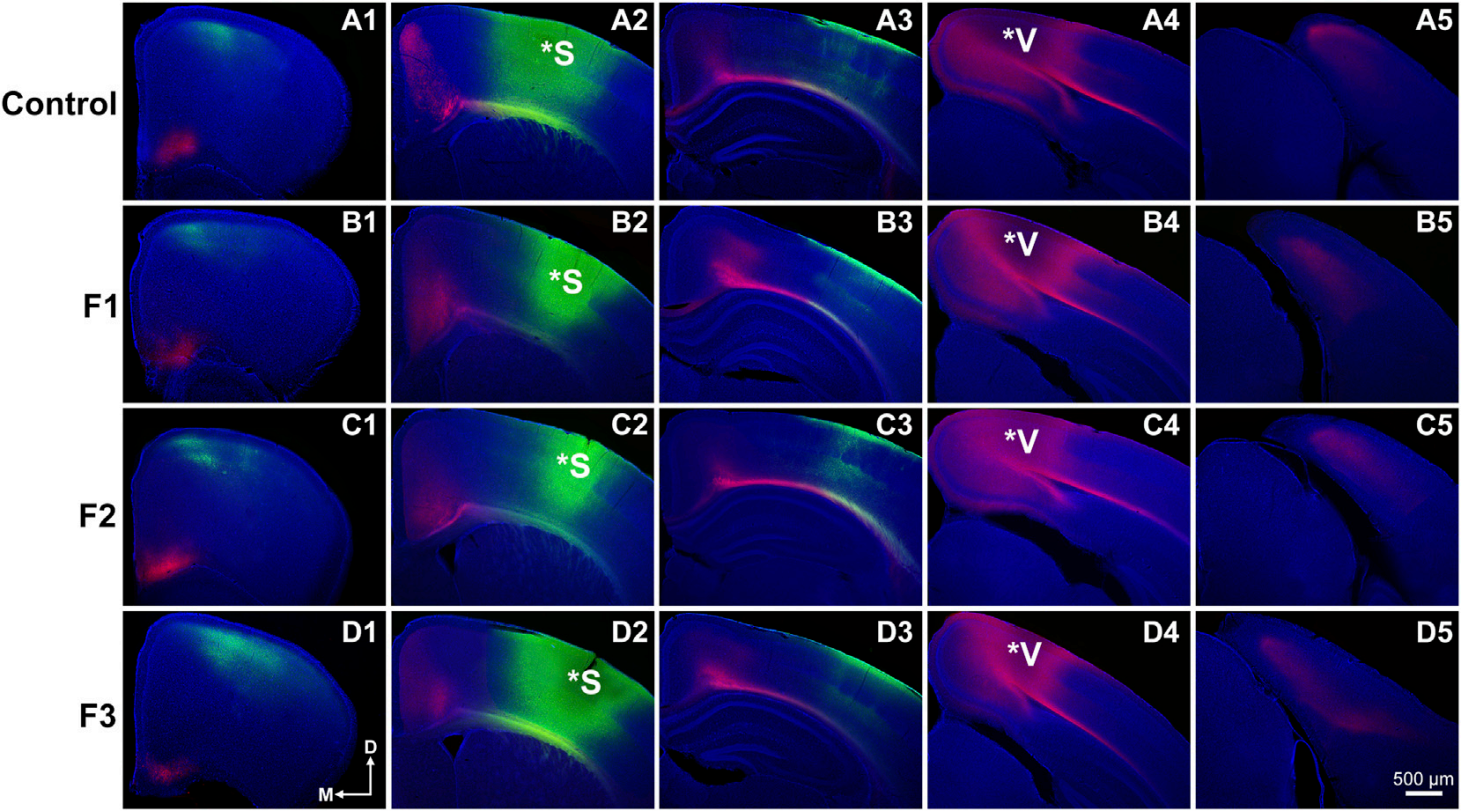
Analysis of primary somatosensory (S1) and primary visual (V1) cortex INC patterns at P20. Rostral-to-caudal series of hemisected coronal sections in individual representative Control (female, **A1-4**) and F1 (male, **B1-4**), F2 (female, **C1-4**) and F3 (male, **D1-4**) P20 mice brains following DiA (green) placement in S1 (*S, **A2-D2**) and DiI (red) placement in V1 (*V, **A4-D4**). No significant phenotypic differences in S1 or V1 projection zones were found between experimental and control brains. All 100 μm sections counterstained with DAPI (blue). Images oriented dorsal **(D)** up and medial **(M)** left. Scale bar, 500 μm.

Precise connectivity of cortical areas is required for the generation of complex behaviors and transgenerational ethanol-induced disorganization of sensory area intraneocortical connections (INCs) are present in newborn PrEE F1-3 mice (Abbott et al., 2018). Here, we examined whether these early-life alterations persist in P20 mice, using lipophilic dye tracing (Figure 4). Single hemispheres from all three experimental generations and control mice were assessed for cortical connectivity via green DiA crystal placements into somatosensory cortex (Figures 4A2–D2) and red DiI crystal placements into visual cortex (Figures 4A4–D4) and patterns of INCs were analyzed across rostral to caudal series of coronal sections. Overall, no distinct differences were found among any of the three experimental generations compared to controls. Typical patterns of prepubescent connectivity were found for somatosensory cortex, including labeled cells within somatosensory subareas and motor cortex in all mice. Visual cortex analysis yielded similar results, with all groups displaying typical connectivity patterns including labelled cells within cingulate, retrosplenial and visual areas. In summary, no discernable changes in sensory cortex connectivity patterns were present in P20 mice due to transgenerational PrEE, suggesting that the drastic disorganization seen in newborn mice (Abbott et al., 2018) is recovered by this age.

### Cortical Gene Expression: *Id2* and *RZRβ*

**FIGURE 5:**
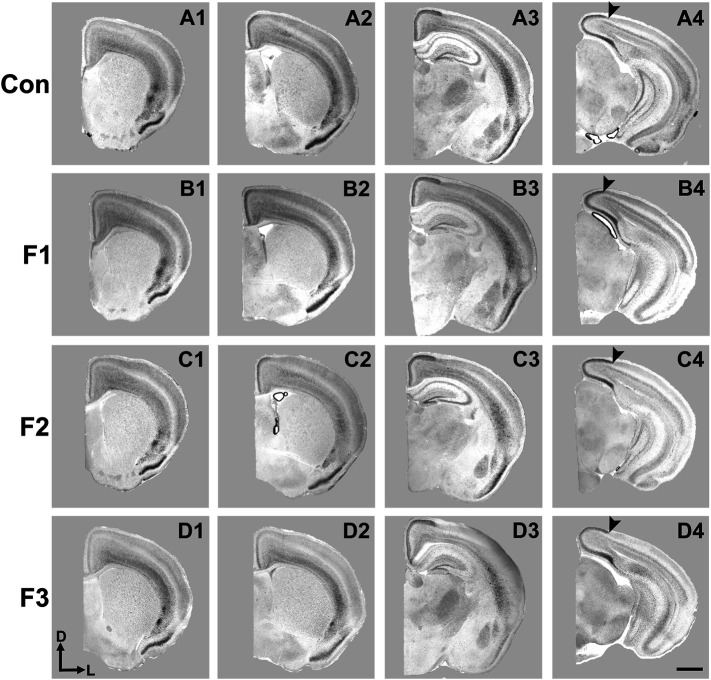
Cortical *Id2* expression at P20. Rostral-to-caudal series of coronal sections following *in situ* RNA hybridization with probes against *Id2* in individual representative control (female, **A1-4**), F1 (female, **B1-4**), F2 (female, **C1-4**), and F3 (female, **D1-4**) mice. F1 mice display a pronounced shift in the lateral limit of the superficial *Id2* band compared to controls (**A4** vs. **B4**, arrows). Images oriented dorsal **(D)** up, lateral **(L)** right. Scale bar, 1 mm.

Spatially and temporally-defined gene expression patterns guide key developmental events within the early postnatal cortex, including axonal targeting, outgrowth and synaptogenesis, which sculpt mature neural circuits. Newborn mice exposed to PrEE display altered expression patterns of key genes for cortical layer and area specification which are also passed transgenerationally, including *Id2* and *RZRβ* (Abbott et al., 2018). Here, we examined expression patterns of these two genes within P20 cortex using *in situ* RNA hybridization in all three experimental generations and controls. Representative F1, F2, F3 and control cases are presented in rostral to caudal order of coronal sections for *Id2* (Figure 5) and *RZRβ* (Supplementary Figure S1).

Expression patterns of *Id2* are highly dynamic across both cortical areas and layers (Rubenstein et al., 1999). Anatomically-matched section analyses revealed largely similar patterns of *Id2* expression at P20 among the F1, F2, F3, and control mice, particularly within rostral and medial sections (Figure 5). Notably in caudal cortical sections, control mice typically displayed a strong, superficial layer expression band that wraps around the medial cortical wall and encapsulates primary visual, secondary visual, and retrosplenial cortices, as previously reported (Dye et al., 2011b). However, in F1 mice, the lateral limit of this expression band is shifted medially and does not extend to the position seen in control mice (Figures 5A4,B4, arrows). In contrast, this lateral limit is phenotypically similar among controls, F2 and F3 cases (Figures 5C4,D4), suggesting that the phenotype (present at P0, Abbott et al., 2018) is rescued by P20 in the F2 and F3 generations.

**FIGURE 6:**
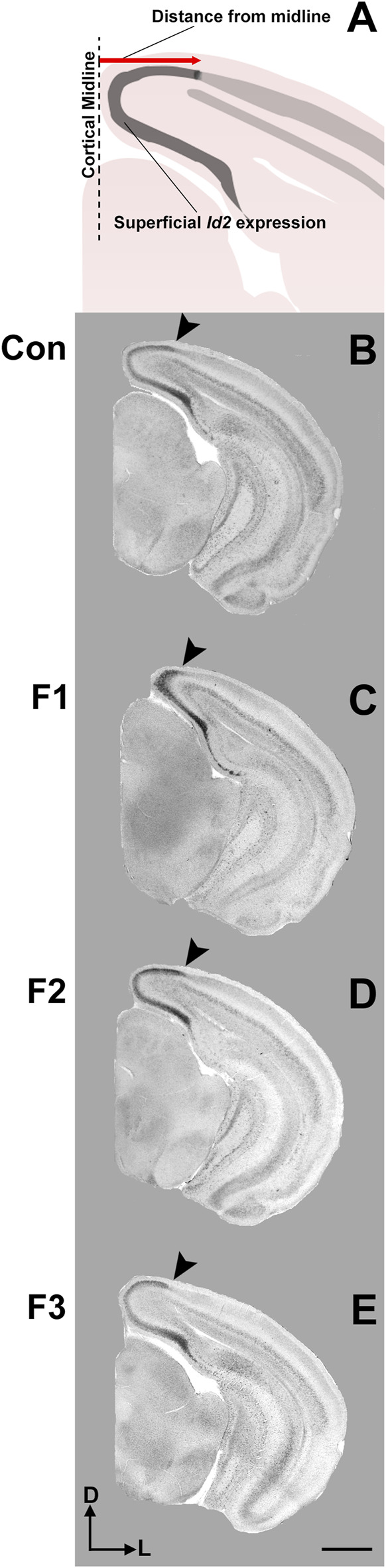
Cortical *Id2* phenotype in F1 mice. **(A)** Line drawing of *Id2* ROI and measurement technique. Individual representative cases are displayed for control mice (male, **B**), F1 mice (male, **C**), F2 mice (female, **D**) and F3 mice (female, **E**). Arrows depict the lateral limit of *Id2* expression, demonstrating a PrEE-induced phenotype in F1 mice. Coronal images of *in situ* RNA hybridization sections oriented dorsal **(D)** up, lateral **(L)** right. Scale bar, 1 mm.

**FIGURE 7:**
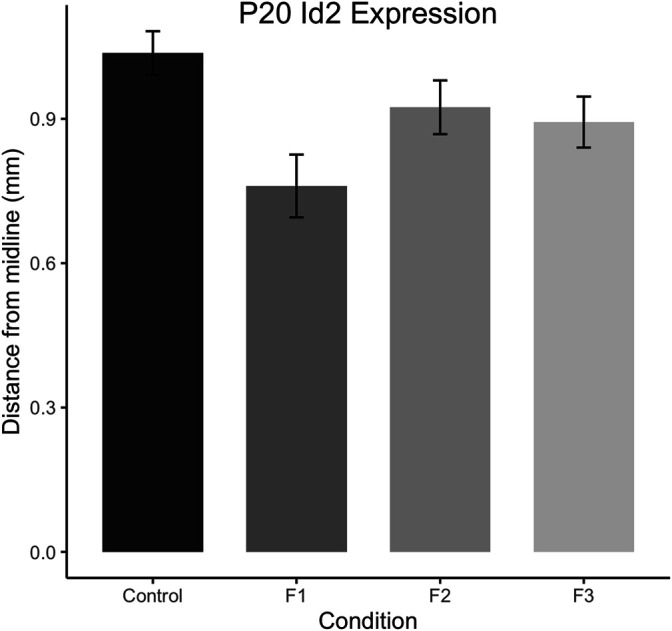
Quantification of neoortical *Id2* phenotype in F1 mice. Distance from the midline to the limit of the robust superficial band of *Id2* expression was calculated for all cases at anatomically-matched sections in Control, F1, F2 and F3 mice at P20. F1 mice display significantly decreased distances from the midline compared to controls (*p* < 0.05).

To confirm this qualitative assessment of *Id2* expression, we utilized a simple measurement from the midline of all cases to measure the lateral limit of this superficial expression band (Figure 6A). One-way ANOVA of these measurements revealed a significant effect of treatment on distance from the midline [F_3,13_ = 3.916, *p* = 0.0341], and post hoc tests revealed that this effect was largely driven by F1 mice, as only the F1 generation was shown to have a significant decrease in this measure compared to controls (control: 1.037 ± 0.0453 mm, F1: 0.7605 ± 0.0652 mm, *p* = 0.0217, Figures 6B,C, 7). Overall, altered expression of *Id2* was found in P20 F1 mice, similar to deficits seen at P0 (Abbott et al., 2018), however F2 and F3 generations (Figures 6D,E) do not display such *Id2* expression differences suggesting that these PrEE-induced changes in P20 persists significantly only in directly-exposed mouse offspring (Figure 7). For *RZRβ*, typical cortical expression patterns were found in all groups including strong layer-specific expression largely localized to layer IV, as well as area-specific patterns including robust somatosensory whisker barrel field expression, suggesting PrEE-induced transgenerational changes in *RZRβ* expression are recovered by adolescence in mice (Supplementary Figure S1).

### Spine Density Analyses Within Primary Somatosensory Cortex

**FIGURE 8:**
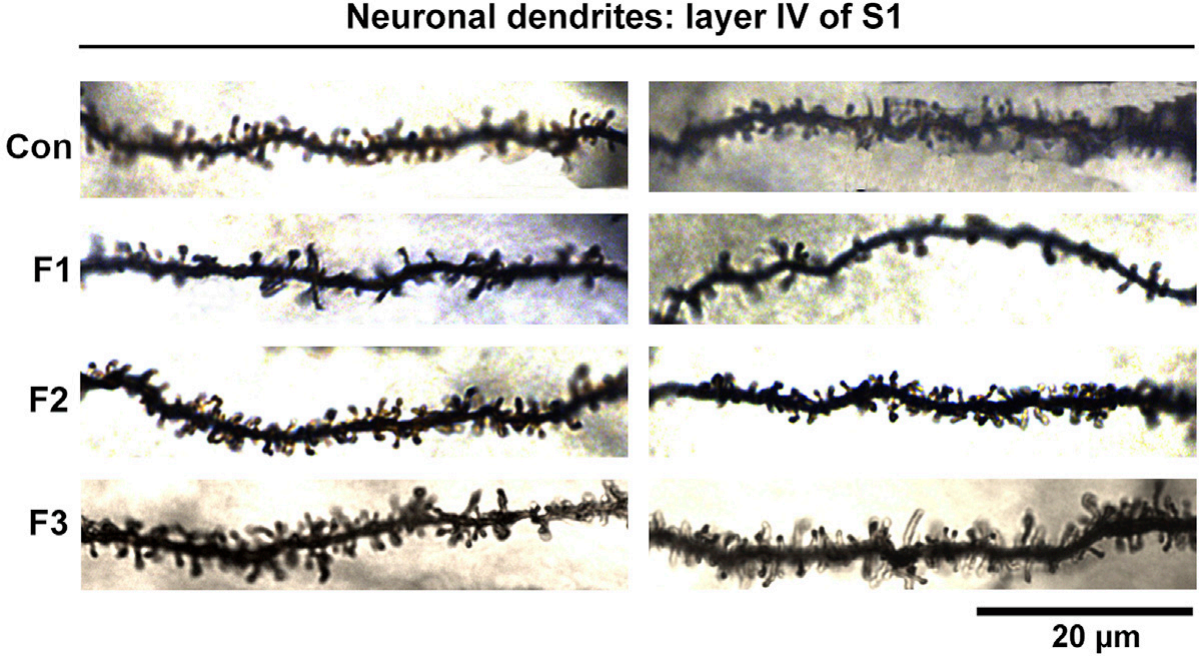
Neuronal Dendrites within layer IV of S1. Two example dendrites are shown for each experimental group originating from separate animals. For Controls: 2 males displayed; For F1: 1 male, 1 female displayed; For F2: 2 males displayed; For F3: 1 female, 1 male displayed. A notable reduction in the number of spines in F1 mice can be observed. Scale bar, 20 μm.

Spine density within cortical areas is associated with sensory processing (Pyronneau et al., 2017) and has also been correlated to alterations in behavioral outputs (Fox et al., 2020). To further uncover potential mechanisms underlying the behavioral dysfunction seen in mice exposed to prenatal ethanol transgenerationally, we investigated neuronal spine density within primary somatosensory cortex (S1). Specifically, spine densities were quantified in spiny stellate cells residing in layer IV of S1 following modified Golgi-Cox staining in F1, F2, F3 and control mice (Figure 8).

**FIGURE 9:**
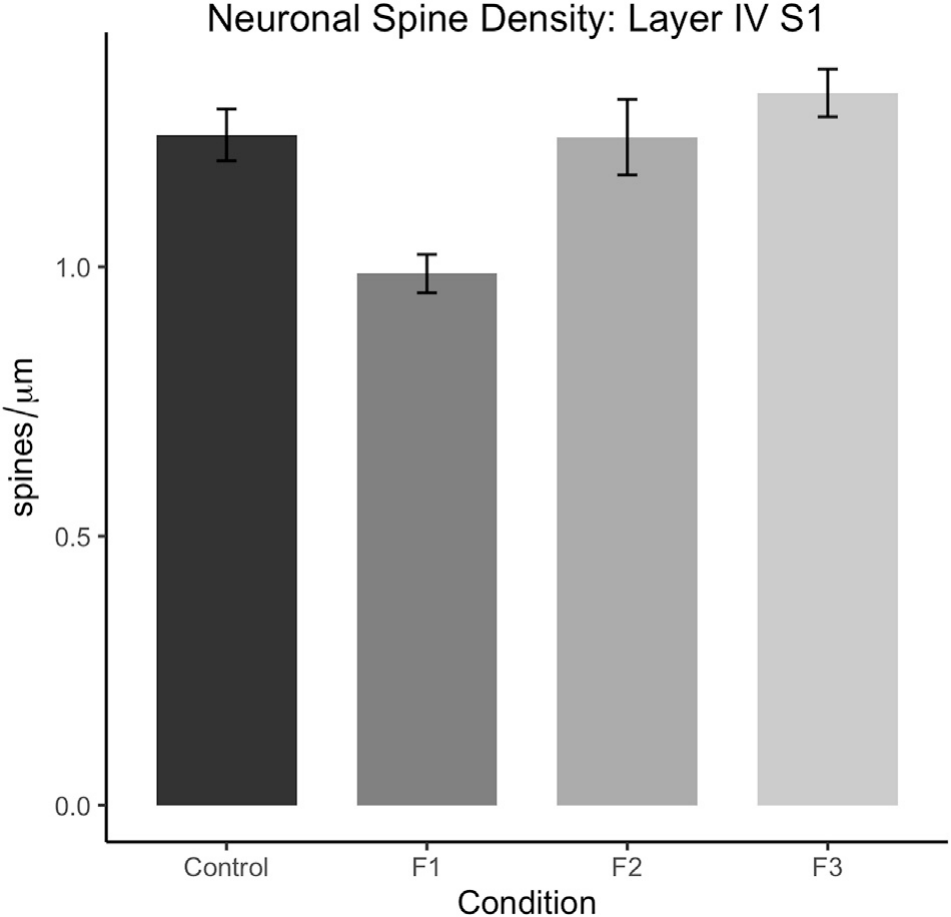
Dendritic spine density analysis in layers IV of spiny stellate neurons in S1 at P20. Spine density (spines/µm) is significantly decreased in S1 spiny stellate cells of F1 mice compared to cells of control mice (*p* < 0.01). Neurons of F2 and F3 mice display no significant differences in dendritic spine density compared to controls.

Following quantification, one-way ANOVA showed a significant effect of treatment on S1 spiny stellate cell spine density [F_3,54_ = 10.19, *p* < 0.0001]. Post hoc tests revealed a significant decrease in spine density in F1 compared to controls (control: 1.245 ± 0.0483 spines/µm, F1: 0.9872 ± 0.0356 spines/µm, *p* = 0.0016; Figure 9). However, no differences were present between F2 or F3 mice compared to controls (compare two sample neurons per condition in Figure 8). This suggests that PrEE-induced reductions in S1 spine density persist to P20 in F1, directly-exposed PrEE mice, but that changes are not apparent in subsequent generations (F2, F3) at P20.

## Discussion

In this study, we demonstrate robust behavioral disruptions in prepubescent mice exposed to prenatal ethanol transgenerationally. F1, F2, and F3 mice display sensory processing deficits, increased risk-taking behavior, as well as increased depressive-like behavior at nearly 3 weeks of age. However, following a multi-level analysis of several key neocortical components including sensory area connectivity, gene expression, and spine density analysis, we found no evidence of heritable, transgenerational phenotypes due to PrEE at P20 within the measures assessed. In contrast, F1 mice display decreased brain/body weights, altered *Id2* expression, and reduced spine density in S1 neurons, providing further insight into the underlying etiology of direct PrEE. Our findings highlight the need for an expanded, in-depth analysis of CNS characteristics which may be driving the stable behavioral phenotypes associated with transgenerational PrEE.

### Transgenerational Impact of PrEE on Prepubescent Behavior

Humans with FASD display sensory processing deficits (Jirikowic et al., 2020), increased risk-taking behavior and poor impulse control (Furtado and Roriz ST de, 2016), as well as increased incidence of depression (O’Connor et al., 2002). Here, we found rodent behavioral correlates that mirror these FASD phenotypes to be present within directly exposed offspring with extension to F2 and F3 generations. In the current report, we show that prenatal ethanol exposure induced several behavioral impairments in F1 mice that persisted to P20 and passed to the second and third (F2, F3) generation. First, we observed a marked impairment in sensorimotor integration and motor performance as measured by the Accelerated Rotarod. In particular, F1, F2 and F3 mice show significant decreases in performance in early trials of this multi-trial assay when compared to controls. However, this effect is recovered in trials 3 and 4 of the task in all three experimental generations, suggesting that these mice are potentially adapting via learning despite an initial, short-term deficit. Interestingly, these results may reflect the ability of task training to improve behavioral outcomes, which has been described in patients with FASD (Nash et al., 2015). Although a similar learning phenomenon may also be expected in control mice, one is likely not present within the current data set due to a possible ceiling effect in which control mice perform at high levels even in early trials.

Evidence for transgenerationally passed behavioral deficits due to PrEE were also found in the Adhesive Removal task, where F1, F2 and F3 mice showed significant impairments in the time before adhesive removal across all three trials compared to controls. These data suggest a substantial impact of PrEE on sensorimotor integration and fine motor processing, mirroring that which is seen in the clinical FASD population (Jirikowic et al., 2020). However, the extension of this clinically-relevant phenotype to the subsequent generations seen here suggests this novel phenomenon may also produce similar effects within the clinical population, which has yet to be fully examined for the potential, transgenerational effects of PrEE.

Similarly, we found strong behavioral change in all 3 PrEE generations in the Elevated Plus Maze, a classical rodent test for anxiety-like behavior. However, while an increase in anxiety-like behavior may be expected due to this well-described effect in humans with FASD, we hypothesize that the increase in time spent on the open arms seen in F1, F2 and F3 mice instead may reflect increased risk-taking behavior. A previous report from our laboratory found that within a classical fear-conditioning test, PrEE mice show a marked decrease in fear memory retrieval to a shock-conditioned tone (Kozanian et al., 2018), suggesting that PrEE may negatively alter perception to aversive stimuli, such as open areas, which mice typically avoid. These potential increases in risk-taking behavior and impulse control have also been described in patients with FASD (Furtado and Roriz ST de, 2016). Additionally, all three experimental generations display increased time immobile in the Forced Swim Test, thought to reflect increased depressive-like behavior in rodents. These results, in combination with the strong transgenerational impacts seen in the Elevated Plus Maze, suggest that psychiatric disorder-relevant behaviors may have the capability to be passed to multiple generations following a single-generation insult.

Although yet to be fully explored within a clinical population, our findings confirm and extend our previous report, where transgenerational inheritance of anxiety-like behaviors and sensorimotor dysfunction was described in several generations of PrEE offspring (Abbott et al., 2018). Our research also adds to the growing collective of transgenerational behavioral effects in rodent models of environmental exposures including morphine exposure (Vassoler and Byrnes, 2021), cocaine exposure (Le et al., 2017) and early postnatal stress (van Steenwyck et al., 2018). Interestingly, despite the presence of several transgenerational behavioral phenotypes presumably from PrEE, F1 mice display increased severity in some of these phenotypes (i.e., within Accelerated Rotarod and Adhesive Removal tests).

Overall, the results described here strengthen the hypothesis that epigenetic processes may play a role in the etiology of several mental disorders (Nestler et al., 2016) and environmental exposures, including PrEE, may carry significant risk of abnormal behavior development for several generations of offspring. Further studies must explore the potential underlying, epigenetic changes that may drive the passing of disease-relevant behaviors to further generations.

### Lack of Evidence for PrEE-Induced Transgenerational Brain Phenotypes in Prepubescent Mice

Despite robust behavioral deficits that extend several generations, our multi-level attempts were unsuccessful in finding any significant transgenerational changes within the neocortex at this age, suggesting that the neurological source(s) underlying the altered behaviors have yet to be found. Previous reports have suggested that PrEE-induced disrupted patterns of sensory cortex INCs in newborn mice may underlie the described behavioral disruptions in adolescence (El Shawa et al., 2013; Bottom et al., 2020), that were also present transgenerationally (Abbott et al., 2018). However, using our methodology, we found no clear transgenerational evidence for grossly disrupted sensory cortical area connectivity patterns due to PrEE, even within the F1 generation, at P20, suggesting the effect observed for all generations at P0 may be recovered by this age. However, as lipophilic dye tracing is not cell or direction-specific (Perrin and Stoeckli, 2000), it remains unclear if any cell-type specific or synapse-specific characteristics within these or a combination of other circuits are altered at P20, and how they may ultimately contribute to the transgenerationally-stable behavioral phenotypes. Additionally, functional deficits, described previously within rodent cortex due to PrEE (Granato et al., 2012; Delatour et al., 2020), may also contribute to the behavioral phenotypes seen in F2 and F3 mice, as proper functional connectivity is essential for complex behaviors.

In this study, we report several novel phenotypes within the directly exposed P20 F1 brains that may be instrumental for the manifestation of FASD-like behaviors. These findings include reduced brain weight and cortical length, altered cortical *Id2* expression, as well as reduced spine density within spiny stellate cells in layer IV of S1; these phenotypes all persist to at least P20. The reported brain size reductions, especially within the neocortex, are associated with several diseases that display cognitive deficits (Hu et al., 2014), likely influencing the behavioral phenotypes of F1 mice. *Id2*, or inhibitor of DNA binding 2, is a transcription factor important for several aspects of CNS development, including axonal outgrowth (Konishi et al., 2004; Huang et al., 2019) and proper positioning of sub-cerebral projection neurons within the neocortex (Maruoka et al., 2011). Although the anatomical difference of *Id2* expression in F1 mice compared to controls is not directly associated with a quantified decrease in mRNA expression (i.e., measured via quantitative PCR), our ISH protocol labels mRNA within the tissue and thus the observed, quantified shift in expression serves as a proxy for a decrease in *Id2* mRNA expression. Further studies should attempt to directly resolve and quantify this anatomically identified difference via more advanced quantitative methods. Overall, our results suggest that these processes, or others, may be disrupted as a result of the consistent, shifted expression patterns of *Id2* in caudal cortex in F1 mice and may contribute to the altered behaviors seen in the same mice.

Spiny stellate cells are a major excitatory neuron class within cortical layer IV which are essential to sensory signal processing (Schubert et al., 2003); here, we show reduced spine density within somatosensory cortex of F1 mice. As proper maintenance of spine density is essential for precise sensory processing (Sweet et al., 2009; Pyronneau et al., 2017), the reduced spine density in the cortex of P20 F1 mice may directly underlie their somatosensory processing deficits observed in the Adhesive removal test data. Spine density has recently been correlated with behavioral outputs (Fox et al., 2020), providing further evidence for this potential underlying phenotype. Collectively, the differences seen in brain weight/size, *Id2* expression and stellate cell spine density may account for, at least in part, to the increased PrEE-induced severity of behavioral disruption in F1 mice discussed above.

### Potential Mechanisms Underlying Transgenerational Inheritance of Behavioral Phenotypes

It is critically important to investigate potential driving mechanisms that may underlie unknown circuit/brain changes and behavioral disruptions within transgenerational PrEE offspring. Recent reports within animal models of PrEE have suggested transgenerational epigenetic inheritance may play a key role in inducing adverse phenotypes in offspring (Govroko et al., 2012; Abbott et al., 2018; Gangisetty et al., 2020). However, this proposed mechanism is still a topic of debate (Horsthemke, 2018), largely due to the vast majority of the mammalian epigenome undergoing resetting in very early development (Xia and Xie, 2020). Despite this, evidence has suggested some epigenetic marks are resistant to resetting and may act as candidates for epigenetic inheritance (Tang et al., 2015). Recently, sperm RNA, histone modification, and DNA methylation has been implicated in epigenetic inheritance of adverse phenotypes (Gapp et al., 2014; Abbott et al., 2018; Beck et al., 2021; Raad et al., 2021), providing credence to this burgeoning mechanistic view on the potential transgenerational impact of environmental exposures. Our laboratory specifically found PrEE-related changes in DNA methylation, an epigenetic mechanism, in newborn murine neocortex that persisted transgenerationally and correlated with neocortical phenotypes at the same age as well as abnormal behavioral phenotypes at P30. Despite our current observation that some neocortical phenotypes recover by P20 in PrEE mice, such as ectopic intraneocortical connectivity, or, like spine density, are present at P20 in F1 mice but rescued in subsequent generations, it is highly likely that the epigenetic modifications impacting the neurobiology of P0 PrEE mice play a role in the behavioral phenotypes observed at P20 (current study) or P30 (Abbott et al., 2018). Whether these phenomena are contributing to the transgenerational effects of PrEE has yet to be fully explored and should remain a key point of interest in future studies.

It is also important to note that PrEE-related phenotypes present in F2, F3 generations presented here were exclusively passed on via the male germline, i.e., only affected males were used to produce the following generation. This model stands in accordance with our previously published report (Abbott et al., 2018) and further supports the substantial impact of PrEE on the male germline. The conceptualization and formulation of this model was informed by studies examining the transgenerational impact of PrEE which found that PrEE produces penetrant phenotypes in subsequent generations at a much greater level when the male germline was used for propagation (Govorko et al., 2012; Gangisetty et al., 2020). Interestingly, these differences may be directly related to the DNA methylation profiles of sperm in affected males (Govorko et al., 2012), providing a mechanistic framework for these observations. EtOH is a known disruptor of several facets of sperm cells (Bielawski et al., 2002; Rompala et al., 2018), and several studies have noted the ability of EtOH-exposed sires to produce altered behaviors in offspring as well (Liang et al., 2014; Conner et al., 2020). In other model systems, male transmission of altered behaviors is also correlated to changes within the profiles of sperm (Gapp et al., 2014). Together, these data suggest that male-germline-driven impacts on progeny via EtOH may be more impactful than previously thought.

Despite mounting evidence that the male germline may be particularly susceptible to transmitting PrEE-related phenotypes to further generations of offspring, it is unknown whether the exposed female germline can produce similar phenotypes in our model. Future studies should address this by examining a broad range of phenotypes in both female- and male germline-produced populations, as well as by further investigating the mechanisms which may underlie the potential differences. Overall, the evidence reported here supports the idea that the male germline may be susceptible to PrEE and may create substantial impact on generations produced via the male germline.

## Conclusion

Here, we provide direct evidence for PrEE-induced transgenerational disruption of clinically-relevant behavioral phenotypes in a mouse model of FASD, including altered sensory-motor processing, increased risk-taking behavior, and increased depressive-like behavior at P20. Although no mechanistic evidence was found for neurological impairments in transgenerationally-exposed mice at this age, we describe several novel phenotypes in directly-exposed F1 mice at P20. These phenotypes include a reduction in body and brain weights and cortical length and altered cortical *Id2* expression, all effects which persist from birth to the prepubescent period in F1 PrEE mice. Also, we found reduced spine density of spiny stellate cells within S1, which is a novel result in our FASD mouse model. Future directions should include in-depth investigations into CNS-based phenotypes which may be driving transgenerational behavioral deficits due to PrEE, including further circuit-based analyses, physiological/functional analyses, as well as analyses of genes important for circuit maturation and maintenance. Further studies should also aim to directly correlate the potential epigenetic mechanisms and the transgenerational inheritance of these detrimental phenotypes. Results from this study support the idea that environmental exposures, like PrEE, can produce drastic effects in future generations of offspring, a still-emerging concept highly important to human health.

## Data Availability

The original contributions presented in the study are included in the article/Supplementary Material, further inquiries can be directed to the corresponding author.
