# Supplementary figures and images for: Transgenerational Effects of Prenatal Ethanol Exposure in Prepubescent Mice
Source: Front Cell Dev Biol. 2022 Mar 21;10:812429. doi: 10.3389/fcell.2022.812429 (PMC8978834; doi:10.3389/fcell.2022.812429)

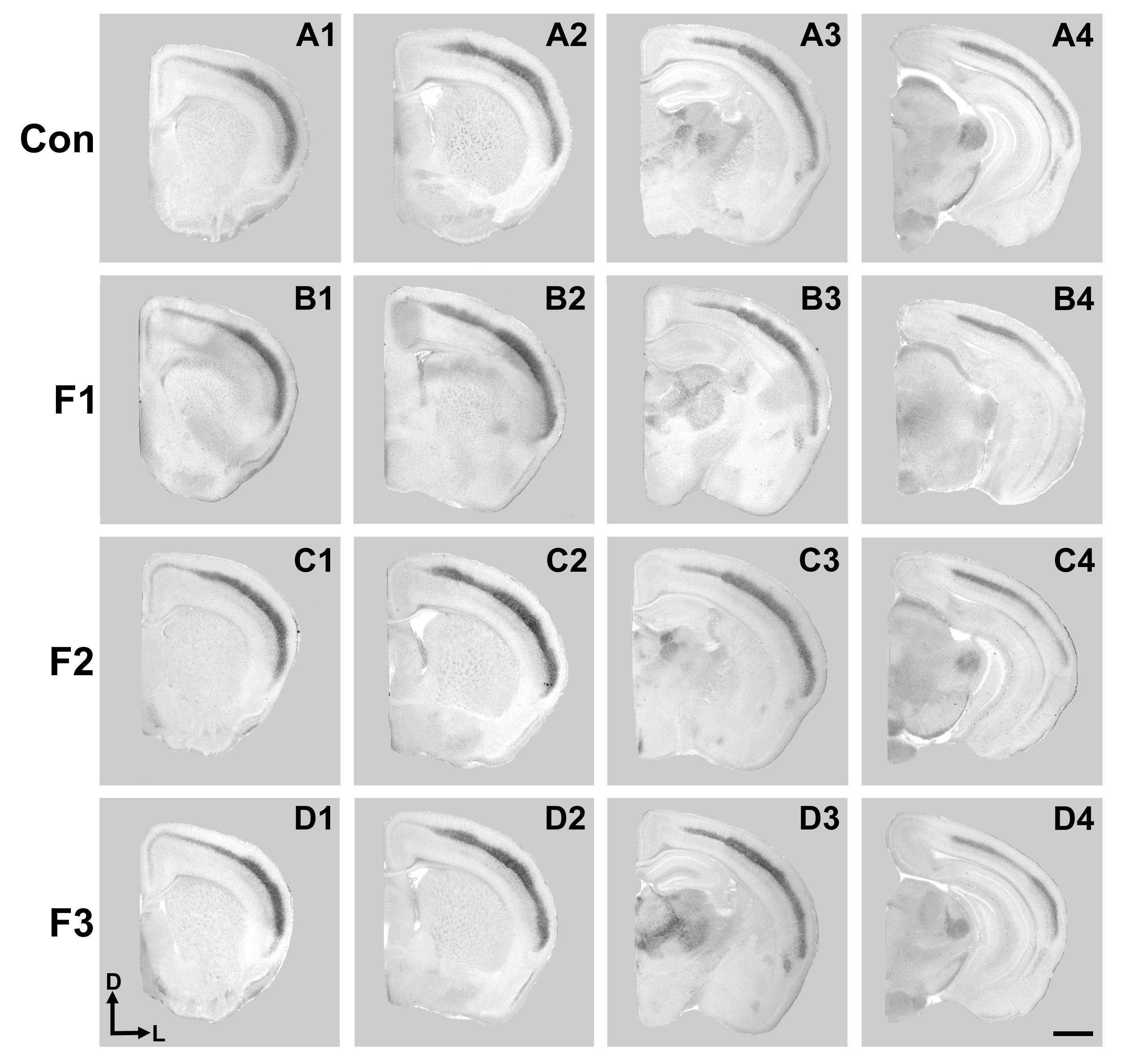

Supplement: Supplementary file 1 [file Image1.tif]
